# A Rare Case of Sheehan Syndrome With Cardiac Tamponade

**DOI:** 10.7759/cureus.24329

**Published:** 2022-04-20

**Authors:** Ahmer Zain, Ashwin Sivakumar, Ozo Akah, Saher T Shiza, Ashwini Mahadevaiah, Aadil Khan

**Affiliations:** 1 General Medicine, Kempegowda Institute of Medical Sciences, Bangalore, IND; 2 Internal Medicine, Angeles University Foundation, Angeles, PHL; 3 Internal Medicine, Carnegie Mellon University, Houston, USA; 4 Internal Medicine, Deccan College of Medical Sciences, Hyderabad, IND; 5 Medicine, Jagadguru Sri Shivarathreeshwara Medical University, Mysore, IND; 6 Department of Surgery, Lala Lajpat Rai Hospital, Kanpur, IND

**Keywords:** sheehan syndrome, pericardial effusion, sella turcica, jaundice, hypopituitarism

## Abstract

Sheehan syndrome, also called postpartum hypopituitarism, is primarily caused by ischemic necrosis of the pituitary resulting from a complicated pregnancy. As the clinical presentations occur years after the complication, it is difficult to diagnose this condition. In this report, we discuss the case of a 35-year-old female with altered mental status, generalized edema, and loss of appetite. The condition was complicated due to the comorbidities of multiple medical conditions such as massive pericardial effusion and untreated jaundice. Her anorexic condition perfectly masked the malnourished appearance of the patient. After multiple laboratory tests and diagnostic imaging, the empty sella turcica of the patient propounded Sheehan syndrome. Replacement of the deficient hormones improved her condition after two weeks. Patients with complicated pregnancy history should be advised for diagnostic imaging early in life to appropriately manage Sheehan syndrome. A delay in diagnosis can have significant health and financial loss. Hormone replacement therapy is the only viable option as there is no cure to treat necrosed pituitary.

## Introduction

Sheehan syndrome, also known as postpartum hypopituitarism, is primarily caused by ischemic necrosis of the pituitary. Necrosis occurs in reduced perfusion of the pituitary caused by childbirth-related hemorrhage. Although postpartum hemorrhage occurs in 3% of women who give birth, Sheehan syndrome is not commonly seen [1]. It is estimated to occur in five out of 100,000 childbirths [2]. Women who give birth in developing countries are at higher risk of Sheehan syndrome due to the increased incidence of postpartum hemorrhage and resource availability to treat hormonal imbalance. Sheehan syndrome manifests as a result of a decrease or absence of one or multiple pituitary hormones. The most affected hormones are prolactin and growth hormone owing to their position in relation to the blood supply of the pituitary. Some of the common predisposing risk factors for Sheehan syndrome include pre-existing vascular conditions for vasospasm and thrombosis, small sella turcica, and other genetic factors. Placental conditions and type 1 diabetes have been known to complicate Sheehan syndrome. Sheehan syndrome has a wide range of symptoms, ranging from mild to severe. Inability to produce milk, fatigue, metrorrhagia, amenorrhea, and hot flashes are a few widely known symptoms [2]. Usually, symptoms appear years after delivery, but they occur acutely and become life-threatening in rare instances. Sheehan syndrome is diagnosed with clinical examination for its symptoms, blood tests for hormones and sugar levels, and magnetic resonance imaging (MRI) of the sella turcica. The difficulty in diagnosing Sheehan syndrome is accounted for by its occurrence long after delivery and its silent nature [3]. Hormonal therapy and monitoring is the current treatment as there is no viable option to cure necrosed parts of the pituitary. In this report, we discuss the case of a 35-year-old female who presented with altered mental status, generalized edema, and loss of appetite. In this case, the diagnosis of this condition was complicated by other non-associated medical conditions.

## Case presentation

A 35-year-old female was brought to the emergency department due to altered mental status and loss of appetite for 10 days. She also complained of fever, irregular menstrual cycles, shortness of breath, weakness and pain in extremities, and dizziness. Her medical history showed admission to the emergency department three years ago for severe iron deficiency anemia with complaints of lower backache, hypotension, headache, failure of lactation, and weight loss. She was discharged after a week with some medication. The patient had two children, a nine-year-old son and a six-year-old daughter. she also had pregnancy loss six years before, following which she developed irregular menses, weakness, and loss of appetite.

On physical examination, the patient had a malnourished appearance with facial puffiness, alopecia, and bilateral edema of the lower extremities, along with a hoarse voice. Her vitals recorded showed that she was hypotensive with raised jugular venous pressure. The patient’s hematological and biochemical parameters are shown in Table 1 and Table 2, respectively.

**Table 1:** Hematological parameters of the patient.

Parameter	Reference range	Day 1	At discharge
Hemoglobin, g/dL	12–16.5	10.5	10
Lymphocytes, %	20–40	14	5
Platelet count, lac cells/mm^3^	1.5–4.5	0.79	0.38
Mean cell volume, fL	80–100	62.2	72.5
Mean corpus hemoglobin, pg	27–32	25.6	23.8
Mean corpus hemoglobin concentration, g/dL	32–35	41.1	32.9
Red blood cell distribution width	11.5–14.5	23.6	21
Red blood cell distribution width absolute volume	37–54	69.8	-
Packed cell volume, %	36–46	25.6	30.6
Neutrophils/mm^3^	1.2–8	6.5–103	11.5–103
Lymphocytes/mm^3^	0.5–5.0	0.6–103	0.4–103

**Table 2:** Biochemical parameters of the patient.

Parameter	Reference range	Day 1	At discharge
Serum sodium, mmol/L	137–150	142.1	137.2
Serum potassium, mmol/L	3.5–5.3	3.58	2.23
Serum calcium, mg/dL	1–5.5	3.6	2.32
Serum total bilirubin, mg/dL	0–1.2	2.7	-
Serum direct bilirubin, mg/dL	0–0.2	1.5	-
Serum indirect bilirubin, mg/dL	0.2–0.7	1.2	-
Serum protein, g/dL	6.0–8.3	6.4	5.8
Serum glutamic-oxaloacetic transaminase, IU/L	<40	74	33
Serum glutamic pyruvic transaminase, IU/L	<34	12	63
Serum alkaline phosphate, IU/L	<240	406	220

**Figure 1:**
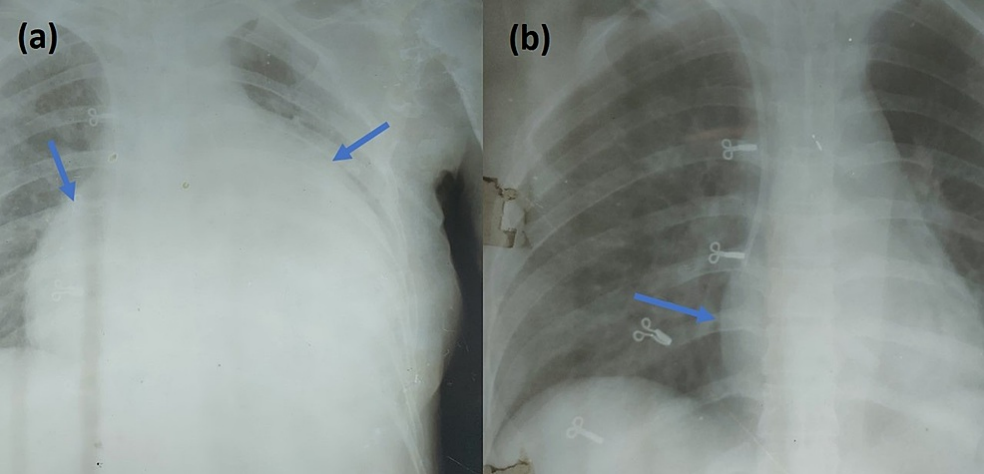
Chest X-ray showing bilaterally enlarged cardiac silhouette (a) and cardiac silhouette after pericardiocentesis (b).

Her chest X-ray revealed a bilateral enlarged cardiac silhouette which raised suspicion for pericardial effusion (Figure 1). Her subsequent echocardiography showed massive pericardial effusion with tamponade physiology dimensions of anterior 1.3 cm, lateral 3.1 cm, and posterior 2.3 cm, along with normal valve and chamber size. The left ventricular ejection fraction was 55%. We performed pericardiocentesis and drained almost 850 mL of fluid. Pericardial fluid microscopy revealed moderate cellularity with few lymphocytes, few polymorphs, and mesothelial cells/macrophages in the background of red blood cells. No malignant cells were seen, and it was negative for any acid-fast bacilli, bacteria, and anti-nuclear antibodies. We ruled out all possible causes of pericardial effusion; however, owing to her finding of facial puffiness, lower extremity edema, and loss of appetite, we suspected hypothyroidism could be because of pericardial effusion. Moreover, the thyroid function test confirmed that she was in severe hypothyroidism (Table 3).

**Table 3:** Hormone levels of the anterior pituitary.

Hormones	Reference range	
Adrenocorticotropic hormone	0–46 pg/mL	Below 5
Prolactin	4.79–23.3 ng/mL	0.689
Thyroid-stimulating hormone	0.35–5.5 uIU/mL	0.63
Free T3	2.30–5.0 pg/mL	1.28
Free T4	12–32 pmol/L	0.82

**Figure 2:**
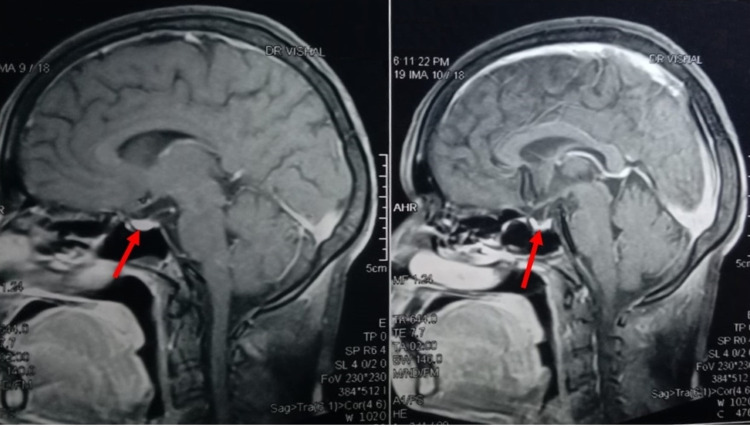
MRI of the mid-sagittal section of the brain showing partially empty sella turcica with a normal stalk (arrows).

After managing most of the immediate concerns, the patient’s vitals started responding to the treatment of hypothyroidism. Based on her thyroid level, irregular menstrual cycle, and the nature of the last pregnancy, an MRI of the head and hormones level of adrenocorticotropic hormone and prolactin were ordered, suspecting Sheehan syndrome. The MRI showed thinning of the pituitary gland with partially empty sella turcica with normal stalk, confirming our suspicion of Sheehan syndrome with below normal anterior pituitary hormone levels (Figure 2). She was started on a drug regimen (Table 4). A follow-up two weeks later showed improved symptoms except for an irregular menstrual cycle but a good prognosis overall.

**Table 4:** Drug regimen of the patient.

Dosage form	Drug	Titration
Tablet	Cefuroxime axetil	250 mg twice a day
Tablet	Esomeprazole	40 mg once a day
Tablet	Iron-folic acid	50 mg + 1.5 mg twice a day
Tablet	Methylprednisolone	4 mg twice a day
Tablet	Vitamin B complex	Twice a day
Tablet	Fludrocortisone	0.1 mg once a day
Tablet	Ondansetron	4 mg three times a day
Tablet	Levothyroxine	25 µg once a day
Syrup	Aptivate	10 mL twice a day
Syrup	Mutivitamin and multimineral	10 mL thrice a day

## Discussion

Although our understanding of Sheehan syndrome has greatly improved since its discovery, a clear understanding of the prognosis and associated risk factors is still a far cry. Due to limited literature and its rarity, Sheehan syndrome is receiving less attention in the medical field. Yet, it is the sixth most frequent cause of growth hormone deficiency and even more frequent in regions with compromised prepartum care [4]. The life-threatening nature of hypopituitarism is evident because when compared with the general population, patients with hypopituitarism have reduced mortality and an increased possibility of experiencing cardiovascular complications [4]. Predicting whether a group of women would develop Sheehan syndrome well before pregnancy involves more speculation as having a postpartum hemorrhage does not completely predispose an individual, and women with a small sella turcica are not relatively safe.

Improvement in obstetric care is expected to yield a better outcome as developed countries have a lower incidence. Developed countries also have lesser diagnostic delays than developing countries. The mean diagnostic delay in a study conducted in France was 9 ± 9.7 years which is six years lesser than the mean diagnostic delay obtained from a similar study in India of 15.35 ± 6.74 years [5,6]. Moreover, the classic underreporting of cases can be seen in the study from India; only 33% of the subjects presented with the typical symptoms of amenorrhea, while 72.2% did not menstruate after the last delivery. It is challenging to record cases with lactational failure; although it is seen in 94.4% of the subjects, no patient showed up with a complaint of isolated lactational failure.

According to a study by Dökmetaş et al. (2006), the most common symptoms included 70% of the patients who lacked postpartum milk production and presented with amenorrhea post-delivery. According to the hormone levels measured in patients, 90% of the patients had secondary hypothyroidism, 55% had an adrenal failure, and almost all of the patients had hypogonadism, prolactin, and growth hormone deficiency. Hyponatremia was present in 35% of the patients [7]. Our patient had amenorrhea, no electrolyte imbalance, and severe secondary hypothyroidism, which caused a pericardial effusion. Hypothyroidism causes pericardial effusion in 3% to 37% of cases and can progress to cardiac tamponade [8].

Our case is noteworthy due to its atypical presentation. Even though some case reports have reported a change in mental status and psychosis as chief complaints of Sheehan syndrome, there is no literature explaining the psychological aspects of Sheehan syndrome [9]. These psychiatric symptoms highly deviate from the typical symptoms. Few pituitary disorders that render the gland non-functional are associated with decreased quality of life and patient well-being [10]. Because there is a deficiency of growth hormone in patients with Sheehan syndrome, studies administering growth hormone replacements for patients presenting with Sheehan syndrome and altered mental status have shown significant improvement in cognitive functioning when measured with an event-related potential device and lipid profile [11]. Finding alternative methods to assess this syndrome clinically may help us diagnose the disease early and provide appropriate treatment. One such ingenious clinical method to screen patients with Sheehan syndrome is to note the prolactin generation response after administering thyrotropin-releasing hormone [4]. Providing hormone therapy early in treatment will prevent the secondary damage caused by Sheehan syndrome. Adding all the newly discovered presentations can help physicians diagnose the condition early. A delay in diagnosis can have significant health and financial loss.

## Conclusions

Neglecting the importance of diagnosing and treating Sheehan syndrome at an earlier stage due to its rarity can lead to unfavorable health and financial outcomes. Diagnosing it early and providing hormone replacement therapy have been shown to reduce the impact of the condition. Approaching the condition with a fixed list of expected presentations will lead the physician in the wrong direction when patients present with diverse symptoms. Our case has shown that patients may present with rare complications such as altered mental status and massive pericardial effusion. Physicians should integrate multiple facets such as pregnancy history and an MRI in diagnosing the condition.
